# Identification of Codon 146 *KRAS* Variants in Isolated Epidermal Nevus and Multiple Lesions in Oculoectodermal Syndrome: Confirmation of the Phenotypic Continuum of Mosaic RASopathies

**DOI:** 10.3390/ijms23074036

**Published:** 2022-04-06

**Authors:** Aude Beyens, Laure Dequeker, Hilde Brems, Sandra Janssens, Hannes Syryn, Anne D’Hooghe, Pascale De Paepe, Lieve Vanwalleghem, Annelies Stockman, Elena Vankwikelberge, Sofie De Schepper, Marleen Goeteyn, Patricia Delbeke, Bert Callewaert

**Affiliations:** 1 Center for Medical Genetics Ghent, Ghent University Hospital, 9000 Ghent, Belgium; aude.beyens@ugent.be (A.B.); sandra.janssens@ugent.be (S.J.); hannes.syryn@ugent.be (H.S.); 2 Department of Biomolecular Medicine, Ghent University Hospital, 9000 Ghent, Belgium; 3 Department of Dermatology, Ghent University Hospital, 9000 Ghent, Belgium; elena.vankwikelberge@uzgent.be (E.V.); sofie.deschepper@ugent.be (S.D.S.); 4 Department of Ophthalmology, General Hospital Sint-Jan Brugge-Oostende, 8000 Bruges, Belgium; laure.dequeker@uzgent.be (L.D.); patricia.delbeke@azsintjan.be (P.D.); 5 Department of Ophthalmology, Ghent University Hospital, 9000 Ghent, Belgium; 6 Department of Human Genetics, University Hospital Leuven, 3000 Leuven, Belgium; hilde.brems@kuleuven.be; 7 Department of Pediatrics, General Hospital Sint-Jan Brugge-Oostende, 8000 Bruges, Belgium; 8 Department of Pathology, General Hospital Sint-Jan Brugge-Oostende, 8000 Bruges, Belgium; pascale.depaepe@azsintjan.be (P.D.P.); lieve.vanwalleghem@azsintjan.be (L.V.); 9 Department of Dermatology, General Hospital Delta Roeselare-Menen-Torhout, 8820 Torhout, Belgium; annelies.stockman@azdelta.be; 10 Department of Dermatology, General Hospital Sint-Jan Brugge-Oostende, 8000 Bruges, Belgium; marleen.goeteyn@azsintjan.be

**Keywords:** epidermal nevus, oculoectodermal syndrome, encephalocraniocutaneous syndrome, nevus psiloliparus, epibulbar dermoid, *KRAS*, RASopathy, non-allelic twin spotting, didymosis

## Abstract

Mosaic RASopathies are a molecularly heterogeneous group of (neuro)cutaneous syndromes with high phenotypical variability. Postzygotic variants in *KRAS* have been described in oculoectodermal syndrome (OES), encephalocraniocutaneous lipomatosis (ECCL) and epidermal nevus syndrome (ENS). This study confirms the continuum of mosaic neurocutaneous RASopathies showing codon 146 *KRAS* variants in an individual with OES and, for the first time, in an individual with (isolated) epidermal nevus. The presence of a nevus psiloliparus in individuals with OES indicates that this finding is not specific for ECCL and highlights the phenotypical overlap between ECCL and OES. The presence of the somatic *KRAS* variant in the nevus psiloliparus resolves the underlying molecular etiology of this fatty-tissue nevus. In addition, this finding refutes the theory of non-allelic twin-spotting as an underlying hypothesis to explain the concurrent presence of two different mosaicisms in one individual. The identification of codon 146 *KRAS* variants in isolated epidermal nevus introduces a new hot spot for this condition, which is useful for increasing molecular genetic testing using targeted gene sequencing panels.

## 1. Introduction

The RASopathies comprise a group of developmental disorders caused by pathogenic variants in genes encoding the RAS/Mitogen-Activated Protein Kinase (MAPK) pathway, including neurofibromatosis type 1, Legius-, Noonan-, cardiofaciocutaneous (CFC)- and Costello syndrome [1]. Mosaic RASopathies, caused by post-zygotic variants in components of the RAS pathway, are an expanding group of (neuro)cutaneous disorders with high phenotypic variability, including for example oculoectodermal syndrome (OES), encephalocraniocutaneous lipomatosis (ECCL) and epidermal nevus syndrome (ENS) [1]. The timing and location of these somatic variants during development determine the extent and distribution of the affected organs. OES is a rare disorder characterized by the consistent combination of congenital scalp lesions and epibulbar dermoids. Aplasia congenita cutis (ACC) is the most common skin finding in OES, characterised by hairless, atrophic and non-scarring regions with asymmetrical distribution [2]. Hamartomas can be associated with the areas of ACC. Other ectodermal findings include linear hyperpigmentation following the lines of Blaschko and, more rarely, epidermal nevus-like lesions. Ocular abnormalities consist of uni- or bilateral epibulbar dermoids, skin tags on the eyelid, and rarely, optic nerve or retinal changes. The phenotype of OES is highly variable and additional features may include growth retardation, cardiovascular manifestations (aortic coarctation, atrial or septal ventricular defects, hypertrophic cardiomyopathy) and mild neurodevelopmental problems (neurodevelopmental delay, learning difficulties and behavioural problems) [3,4]. Patients are predisposed to develop age-dependent benign tumour-like lesions, such as non-ossifying fibromas of the long bones and giant cell granulomas of the jaws [5]. OES shows considerable phenotypic overlap with encephalocraniocutaneous lipomatosis (ECCL), sharing symptoms such as focal alopecia, epibulbar dermoids, linear hyperpigmentation, cardiovascular manifestations and the predisposition to develop benign skeletal tumours [3,5,6]. However, ECCL usually involves the central nervous system, characterised by structural anomalies of the brain and spine, epilepsy, and more pronounced neurodevelopmental delay. So far, the presence of nevus psiloliparus, a smooth fatty tissue nevus with non-scarring alopecia, has been considered pathognomonic for ECCL. Exome sequencing of affected tissue identified somatic mosaicism for *KRAS* variants as a common genetic etiology for OES and ECCL [3,5].

Another heterogeneous group of disorders that belongs to the group of mosaic RASopathies includes epidermal nevi (EN) and their associated syndromes, such as linear nevus sebaceous (NS) syndrome (synonym: Schimmelpenning-Feuerstein-Mims syndrome), phacomatosis pigmentokeratotica and cutaneous-skeletal-hypophosphatasia (CSHS) syndrome. Isolated EN can be subdivided into non-organoid keratinocytic nevi (KEN) or organoid nevi (including NS and nevus comedonicus (NC), according to their predominantly keratinocytic or adnexal differentiation, respectively [7,8]. Isolated NS, KEN and linear NS syndrome are mostly associated with pathogenic variants in *HRAS*, and to a lesser extent with somatic *KRAS* variants [9]. Considering the observed overlap in underlying genotype, body-site specific determinants through distinct epithelial-mesenchymal interactions are thought to contribute to phenotypic diversity [10]. In this study, we confirm the continuum of mosaic (neuro)cutaneous RASopathies as we report on codon 146 *KRAS* variants in an individual with OES and nevus psiloliparus and, for the first time, in an individual with isolated EN.

## 2. Results

### 2.1. Clinical Findings

**Figure 1:**
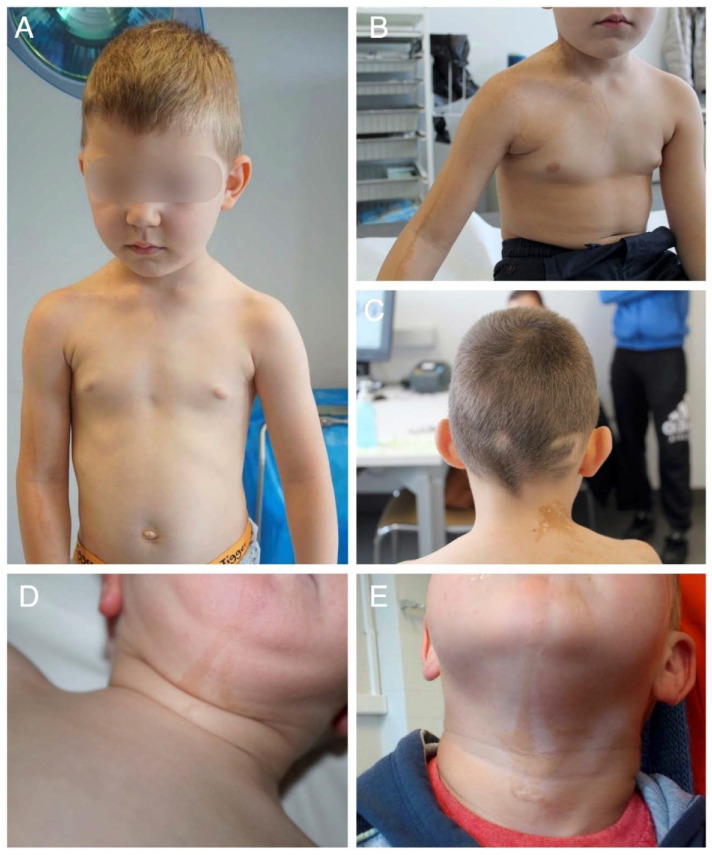
(**A**–**E**) Clinical features. (**A**,**B**) Eyelid coloboma, epibulbar dermoid and segmental areas of hyperpigmentation following a Blaschkoid distribution on the right upper half of the body and extending to the right arm in patient 1. (**C**) The same patient also presents with three areas of focal alopecia with yellow skin colour. (**D**,**E**) Palpable verrucous hyperpigmentation following Blaschko lines on the chin, extending to the neck in patient 2 ((**D**) age 2, (**E**) age 4).

The first patient was a 5-year-old boy referred to our clinic because of the presence of ocular abnormalities, Blaschkoid hyperpigmentation and focal alopecia. He was the only child of healthy, non-consanguineous parents of Romanian descent. He presented with segmental areas of hyperpigmentation following a Blaschkoid distribution on the right upper half of the body and extending to his right arm. The lesions were slightly palpable on his neck. Similar abnormalities on the posterior part of the neck displayed a more yellowish-orange colour. The scalp showed three areas with yellow skin colour, diminished hair density and fair, almost white hairs. Despite the lack of palpable lesions, these skin abnormalities were suggestive of a linear epidermal and sebaceous nevus, respectively (Figure 1A–C). ACC was not present. Ocular abnormalities included a congenital coloboma of the right eyelid and an epibulbar dermoid involving both eyes, for which surgical correction was performed. Additional inspection revealed multinodular tissue that extended to the corneal limbus and covered parts of the visual axis. There was increased fat accumulation in the right upper eyelid (Figure 1A). Strabismus with amblyopia of the left eye was present because of high astigmatism. The patient’s vision improved following correction of the astigmatism with glasses and occlusion therapy.

Additional investigations consisted of a normal cardiac echocardiography, an electrocardiogram, an audiometry screening and a skeletal survey of the hips and lumbar spine. No structural intracranial abnormalities were identified on the brain MRI. His neurocognitive development was normal and he had no other health problems.

The second patient, a 3-year-old boy, was born at term after an uncomplicated pregnancy to non-consanguineous parents. The mother had high myopia with a risk for retinal detachment and some Marfanoid features, but had a normal *FBN1* sequencing. The couple’s first pregnancy resulted in a miscarriage at 7 weeks gestational age. A maternal uncle showed isolated delayed language development, which normalised later in life. In addition, a maternal great aunt presented with mild intellectual disability and worked in a sheltered workshop. The proband presented at birth with a palpable verrucous hyperpigmented lesion following Blaschko lines on the chin and extending to the neck, suggestive of epidermal nevus. The EN showed a discrete elevated surface, but no papules (Figure 1D,E). Upon clinical examination, a hypopigmented macula was observed in the right inguinal area, as well as some linear hyperpigmentation in the knee fold. A small café-au-lait macula was located on the back of the right calf. A further inspection of skin, mucosa, hair and nails was normal, and there was no evidence of ACC or other scalp hamartomas. The patient’s early motor development was borderline with sitting and walking independently at the ages of 8 and 18 months, respectively. However, his active language development was delayed with his first words spoken at the age of 2. His passive language skills were normal. A re-evaluation at the age of 3 years and 9 months showed normal active language skills, normal behaviour and an average IQ. A brain MRI and an ophthalmological evaluation using a slit-lamp examination and a fundoscopy were normal.

### 2.2. Histological Findings

**Figure 2:**
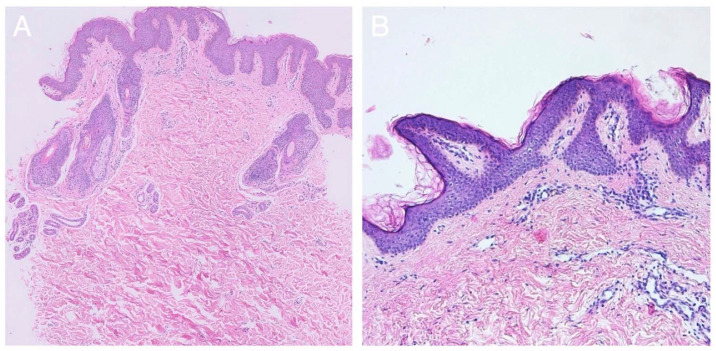

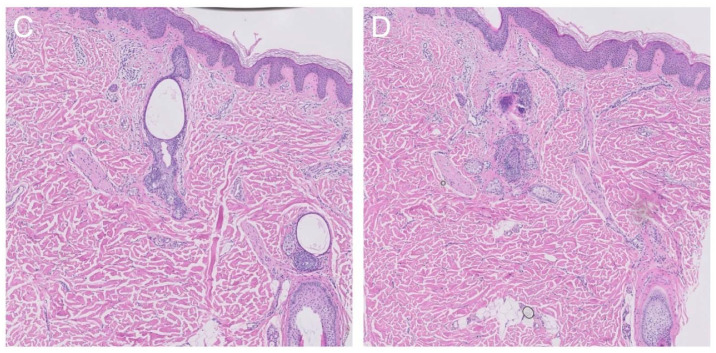
(**A**–**D**) Histopathology. (**A**) A skin biopsy from the slightly palpable Blaschkoid hyperpigmentation on the right arm in individual 1 shows features suggestive of epidermal nevus, including discrete acanthosis, hyperkeratosis and pigmentation of the basal layer (magnification ×100). (**B**) Similarly, epithelial hyperplasia and papillomatosis confirm the diagnosis of epidermal nevus on the chin of individual 2 (magnification ×400). (**C**,**D**) A skin biopsy taken in the focal region of alopecia in individual 1 shows the presence of fat in the dermis, tissue fibrosis and an anagen arrest of hair follicles, indicative of nevus psiloliparus (magnification ×100).

Two different skin lesions were biopsied in individual 1: the linear hyperpigmentation on the right arm, suspicious for linear EN, and the area of focal alopecia on the scalp, suspicious for NS. In the first lesion, discrete acanthosis, hyperkeratosis and pigmentation of the basal layer were observed, which indeed are suggestive of the diagnosis of KEN (Figure 2A). In the second lesion, no typical characteristics of NS were withheld. However, the presence of fat in the dermis, tissue fibrosis and an anagen arrest of hair follicles was found, matching the diagnosis of nevus psiloliparus (Figure 2C,D).

In individual 2, a skin biopsy from the lesion in the neck showed papillomatosis with accompanying seborrheic epithelial hyperplasia and the presence of two inclusion cysts, suggestive of the diagnosis of EN (Figure 2B).

### 2.3. Molecular Genetic Testing

**Figure 3:**
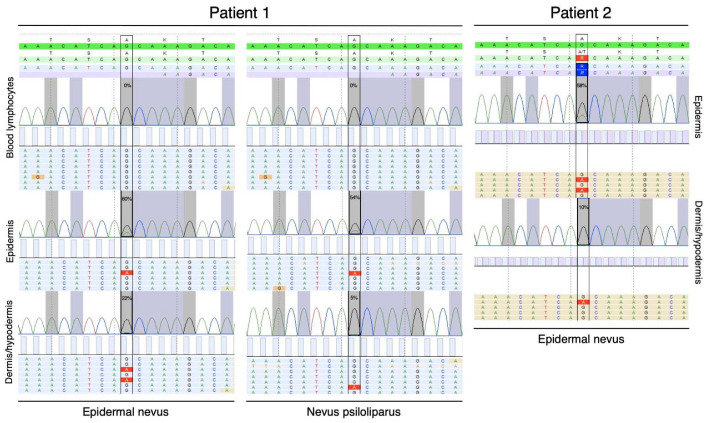
Molecular genetic testing. Left panel: Next-generation sequencing on DNA extracted from a skin biopsy in the epidermal nevus of patient 1 identified a mosaic missense variant (c.436G > A, (p.Ala146Thr)) in the *KRAS* gene with variant frequencies of 60% in cells of the epidermis and 22% in the dermis/hypodermis. DNA extracted from the nevus psiloliparus identifies the same *KRAS* variant with a frequency of 54% in the epidermis and 5% in the dermis/hypodermis. DNA from peripheral leukocytes does not contain the *KRAS* variant. Right panel: Identically, in patient 2, the p.(Ala146Thr) variant was identified in the DNA extracted from the epidermal nevus with a frequency of 58% and 10% in the epidermis and dermis/hypodermis, respectively (green color: reference sequence, blue and brown color: sample sequence, red: aberration from the reference sequence).

Molecular genetic testing in proband 1 comprised molecular karyotyping on DNA isolated from peripheral leukocytes, which showed a paternally inherited 620 kb duplication on the Y chromosome and was classified as benign. Subsequent next-generation sequencing analysis on DNA directly extracted from a skin biopsy taken in the linear hyperpigmentation was able to identify a mosaic missense variant (c.436G > A, p.(Ala146Thr)) in the *KRAS* gene, illustrated by variant frequencies of 60% in cells of the epidermis and 22% in the dermis/hypodermis. This confirms the diagnosis of oculoectodermal syndrome (Figure 3). Additionally, DNA extracted from the nevus psiloliparus was analysed and confirmed the presence of the same *KRAS* (c.436G > A, p.(Ala146Thr)) variants, with a frequency of 54% in the epidermis and 5% in the dermis/hypodermis (Figure 3).

In proband 2, next-generation sequencing analysis on DNA directly extracted from a skin biopsy taken from the EN identified the same mosaic missense variant (c.436G > A, p.(Ala146Thr)) in *KRAS* as identified in the first patient, with a frequency of 58% in the epidermis and of 10% in the dermis/hypodermis. Molecular karyotyping on DNA from peripheral leukocytes identified a maternally inherited 140 kb duplication in chromosome band Xp11.22. This duplication includes the two first exons of the *HUWE1* gene, containing the 5′-untranslated region (5′-UTR). *HUWE1* is associated with X-linked neurodevelopmental and intellectual disability [11]. The variant did not segregate from the neurodevelopmental phenotype in the family and was considered a class 3 variant.

## 3. Discussion

Mosaic RASopathies are an expanding group of pleiotropic disorders caused by postzygotic, activating variants in highly conserved RAS oncogenes and other associated genes of the RAS-MAPK pathway. Variants leading to over-activation of RAS-MAPK signalling are similar to the somatic driver variants observed in these proto-oncogenes during tumorigenesis [12]. As these oncogenic variants are not tolerated in the germline and most often result in embryonal lethality, RASopathy disorders tend to be caused by hypomorphic variants associated with less over-activation of the pathway [13]. There are a few exemptions to this rule, with reported germline oncogenic changes in *HRAS* in Costello syndrome, in *KRAS* in CFC syndrome, and in *PTPN11* in Noonan syndrome [14,15,16]. Codon 146 oncogenic *KRAS* variants have been identified as a common genetic etiology in OES and ECCL, whereas codon 13 and 19 only have been associated with OES [3,5]. Correspondingly, both disorders demonstrate considerable phenotypic overlap, sharing focal alopecia, epibulbar dermoids, linear hyperpigmentation, and a predisposition to develop benign skeletal tumours. Differentiation between the two entities can be challenging but is important due to differences in prognosis and management. ECCL is considered the severest form within the spectrum, characterised by structural brain abnormalities, seizures and intellectual disability [17]. This study confirms the continuum of mosaic neurocutaneous RASopathies due to codon 146 *KRAS* variants. Both reported individuals present with slightly palpable linear hyperpigmentation following Blaschko lines, histopathologically confirmed as KEN. Individual 1 further presented with focal alopecia, congenital coloboma and epibulbar dermoid in absence of any neurological manifestations, suggestive of OES. Interestingly, focal areas of alopecia are suggestive of nevus psiloliparus, rather than aplasia cutis. Nevus psiloliparus, a smooth, hairless fatty tissue nevus on the scalp, has been considered as a pathognomonic hallmark of ECCL, but has also been described in one case of OES and two non-syndromic cases [3,18,19]. As such, this observation further supports the phenotypical overlap between both disorders and indicates that nevus psiloliparus is not solely specific for ECCL.

In individual 2, KEN occurred as an isolated feature. Activated RAS variants can be identified in isolated KEN in 39% of cases, with almost 90% due to hotspot variants in *HRAS*. Despite having been described more frequently in isolated nevus sebaceous and Schimmelpenning syndrome, a sole case of isolated KEN attributed to somatic *KRAS* variants has been reported until now. In both isolated and syndromic forms, the *KRAS* hotspot is located at codon 12 [9,10]. Here, we describe the first case of isolated KEN due to postzygotic codon 146 *KRAS* variants. Since molecular testing for epidermal nevi and their associated syndromes is often restricted to targeted gene analysis of hot spots, we recommend full gene coverage in case hotspot analysis reveals no pathogenic variants. In addition, adequate tissue sampling and processing is of utmost importance to increase the diagnostic yield. Because of the presence of a class 3 *HUWE1* variant, it remains speculative if the mosaic *KRAS* variant is (partly) contributing to an initial delay in language delay development [11]. Nevertheless, the proband’s neuromotor development was able to catch up well.

Historically, the theory of non-allelic twin spotting or didymosis has been put forward to explain the concurrent presence of two different mosaicisms in one individual, as for example in OES with didymosis aplasticopsilolipara (coexistence of aplasia cutis and nevus psiloliparus) or in phacomatosis pigmentokeratotica (PPK) with didymosis spilosebaceus (coexistence of sebaceous nevus and nevus spilus) [20,21]. The hypothesis stated that the occurrence of a recombinational somatic event, by which two non-allelic recessive variants homozygously concur in neighbouring cells, gives rise to the existence of the two observed phenotypes [21,22]. However, the theory has been refuted in PPK by the identification of postzygotic variants in *HRAS* [23,24]. In this report, individual 1 presents with coexistent epidermal nevus and nevus psiloliparus. We identified postzygotic *KRAS* variants in both lesions, which again counters the theory of twin spotting in OES and other mosaic disorders. In addition, this resolves the underlying molecular etiology of nevus psiloliparus, which was hereto unknown.

The pleiotropic and variable phenotype of mosaic *KRAS* variants is primarily related to the timing during development, which determines the extent and distribution of manifestations. As such, body site is thought to be an important element of observed features, reflected by NS occurring mostly on the head and neck, and KEN occurring on other body sites [10,25]. In addition, genotype-determined functional consequences may influence tissue-specific cell fate decision and hence, observed tissue-specific manifestations [3]. RAS functions as a molecular switch between an active GTP-bound form and an inactive GDP-bound form. The conversion is mediated by the intrinsic GTPase activity of RAS [26]. Impairment of this hydrolytic reaction and constitutional RAS activation is the most common biochemical alteration observed in oncogenic RAS variants, which includes codon 12. Contrarily, codon 13 and 146 variants increase nucleotide exchange kinetics due to specific spatial relationships to the RAS nucleotide binding cleft in the absence of increased GTPase activity [27,28]. These functional differences could result in divergent phenotypic manifestations. However, the identification of codon 146 *KRAS* variants in KEN contradicts this hypothesis and suggests that other molecular mechanisms, in addition to the temporospatial distribution of the postzygotic variant, contribute to variability in the clinical spectrum of mosaic RASopathies. Finally, the risk of malignancy in OES/ECCL has been attributed to the presence of oncogenic *KRAS* variants, including giant cell tumours and embryonal rhabdomyosarcoma, which should justify regular clinical follow-up [29,30,31]. Whether this risk for malignancy is also applicable in isolated lesions remains a matter of debate due to the limited number of reported cases.

In conclusion, we reported on codon 146 *KRAS* variants in an individual with OES and, for the first time, one with isolated EN, thereby conforming the continuum of mosaic neurocutaneous RASopathies. We identified codon 146 as an additional hot spot for KEN and resolved the underlying molecular etiology for nevus psiloliparus, refuting the theory of non-allelic twin spotting in mosaic disorders. 

## 4. Materials and Methods

### 4.1. Consent

The legal guardian of the patients gave written informed consent for publication of the case details. Specific informed consent was obtained for the publication of clinical pictures. This study was conducted in accordance with the 1984 Declaration of Helsinki and its subsequent revisions.

### 4.2. Molecular Analysis

Molecular genetic testing consisted of array CHG molecular karyotyping on DNA isolated from peripheral leukocytes using an 180k Agilent array with mean genome-wide resolution of 108 kb (AMADID#022060, hg19/CRCh37, February 2009). Next-generation sequencing analysis on DNA directly extracted from the epidermal and dermal/hypodermal fraction was performed using a tumour hotspot gene panel (Tumor Hotspot MASTR Plus, Agilent, Santa Clara, CA, USA), including *AKT1*, *ALK*, *BRAF*, *CDKN2A*, *CTNNB1*, *DDR2*, *EGFR*, *ERBB2*, *ERBB4*, *FGFR2*, *FGFR3*, *H3F3A*, *HIST1H3B*, *HRAS*, *IDH1*, *KIT*, *KRAS*, *MAP2K1*, *MET*, *NRAS*, *PDGFRA*, *PIK3CA*, *PK3R1*, *PTEN*, and *STK11*. Molecular analysis was conducted at gDNA level using multiplex PCR amplification. The obtained library preps were analysed following the Illumina sequencing by synthesis (SBS) technology (Miseq, Illumina, San Diego, CA, USA), with minimal expected coverage depth of 500×. Obtained sequence profiles were aligned to the respective reference sequences using the SeqNext software version 5.2.0 (JSI Medical Systems GmbH, Ettenheim, Germany). Pathogenic variants were reported using the HGVS nomenclature (www.hgvs.org, accessed on 18 February 2021).

## Data Availability

Not applicable.
